# An Account of the Good Effects of Electricity in a Case of Violent Spasmodic Affection

**Published:** 1792

**Authors:** George Wilkinson

**Affiliations:** Surgeon at Sunderland, and Member of the Royal College of Surgeons at Edinburgh, &c.


### VIII. *An Account of the good Effects of Electricity in a Cafe of violent fpafmodic Affection. By Mr.* George Wilkinfon, *Surgeon at Sunderland, and Member of the Royal College of Surgeons at Edinburgh, &c.*

ON the 25th of May, 1788, I was defired to vifit Mifs A. Crawford, of this place, aged aged twenty-eight years, and of a delicate conſtitution, who had for about three weeks been afflicted with a violent ſpaſmodic complaint. I found her labouring under great dejection of ſpirits; her pulſe was weak, but regular; her appetite was much impaired, yet ſhe had little or no ſickneſs or thirſt: ſhe complained of a ſenſe of weight in her forehead, with dimneſs of ſight, and of a pain about the region of her ſtomach; ſhe was coſtive; her urine, which was much increaſed in quantity, was ſometimes limpid, but in general of an amber colour, and ſhe frequently complained of coldneſs, particularly in her extremities.

On farther inquiry, it appeared that ſhe had enjoyed a tolerable ſhare of health till the 7th of May, when, after much uneaſineſs of mind, ſhe was ſeized at two o'clock in the morning, while in bed, with a rigidity and ſtiffneſs of the whole body. At four ſhe became totally inſenſible, and continued ſo till eight the ſame morning, when, after ſeveral deep ſighs, the fit went off, her limbs became relaxed and her ſpeech and recollection gradually returned.

She had no more attacks of this kind till about a week afterwards; and then they differed greatly in their violence and duration, reſembling hyfteric affections, frequently coming on twice or thrice in the fpace of an hour, and continuing only a few minutes at a time. It often happened that they left her for the fpace of a day, and fometimes for two or three days, but when this was the cafe they returned with greater feverity, more efpecially at the approach of the catamenia, (which were always regular) and previoufly to the coming on of rain or ftormy weather, obferving no regular periods with refpect to their attack, and varying in their duration from fifteen minutes to two hours or longer.

There was no criterion whereby fhe could afcertain the approach of thefe fits, their acceffion being always fudden. An univerfal fpafm, producing a rigidity of the whole frame, took place in a moment, and deprived her of the power of fpeech and recollection. Indeed the frequent opportunities I had of feeing her in thefe fituations enabled me to obferve the progrefs of the fymptoms with attention, and her cafe ftruck me as bearing a ftriking refemblance, in many refpects, to that of a young lady defcribed by a late ingenious and refpectable writer.

" She

"She exhibited," ſays he, "a figure of

"death-like ſleep, beyond the power of art to

"imitate, or the imagination to conceive. Her

"forehead was ſerene, her features perfectly

"compoſed. The paleneſs of her colour, her

"breathing at a diſtance being alſo ſcarce per-

"ceptible, operated in rendering the ſimilitude

"to marble more exact and ſtriking. The

"poſition of her fingers, hands, and arms,

"was altered with difficulty; but they preſerved

"every form of flexure they acquired *."

Previouſly to my attendance ſhe had taken ſeveral medicines, ſuch as valerian, and the fœtid gums in various forms; from theſe, however, ſhe had experienced little or no benefit.

The intention I had in view was to obviate the attack of the fits, by diminiſhing the irritability of the ſyſtem, and to reſtore the ſtomach to its natural functions; for this purpoſe I recommended that, on the acceſſion of the fits, her hands and feet ſhould be immerſed in warm water, and a draught compoſed of vin. antim. æther vit. and tinct. opii, in a ſuitable vehicle, to be taken at proper intervals, varying the doſe

* *Vide* Select Caſes by John Jebb, M.D. 8vo. London, 1782, p. 64.

 of of the ſeveral ingredients according to the urgency of the ſymptoms.

By this treatment the ſtrength and frequency of the fits were diminiſhed.

In the intervals, or abſence of the fits, ſhe took, at proper intervals, as tonics, extract of gentian and rhubarb, with ſalt of ſteel. From the uſe of theſe remedies ſhe experienced great benefit: her appetite improved, and the fits left her for a week.

Bark in ſubſtance, to the quantity of two ſcruples three times a day, being afterwards adminiſtered, diſagreed with her ſtomach, and ran off by the inteſtines, though combined with cinnamon in powder, and with ſmall doſes of tincture of opium.

June 7th, her fits again returned, and continued to attack her more frequently, and with increaſed violence, till the 13th, when I was ſent for in great haſte, and found her ſitting in her chair, perfectly ſenſible, but with her jaw completely locked, her face ſomewhat diſtorted, her head drawn backwards, and the muſcles of her neck rigid and inflexible. The approach of this fit had been preceded, as ſhe informed me, by a ſenſation like the cramp in the ſoles of her feet, ſucceeded by a coldneſs of the extremities, and a ſpaſmodic affection of the muſcles of her neck and jaw.

Under theſe circumſtances, her hands and feet were immerſed in warm water; her neck and throat were repeatedly rubbed with a liniment compoſed of volatile alkali, mixed with ol. terebinth.; a fœtid clyſter, with the addition of a drachm and a half of tincture of opium, was likewiſe adminiſtered; and from thirty to forty drops of the latter, mixed with an equal quantity of æther, were directed to be given every two or three hours in a ſuitable vehicle.

This plan of treatment was purſued till the next morning, when, upon finding her no better, I determined to try the effects of electricity.

Mr. Martin, of this place, who has applied himſelf, with great ſucceſs, to medical electricity, having favoured me with his aſſiſtance, we placed the patient at firſt in an inſulated chair connected with a pretty powerful machine, the cylinder of which was eleven inches in diameter; ſtrong ſparks were taken from various parts of her body, but particularly from the muſcles of the neck: theſe proving ineffectual, ſeveral ſmart ſhocks, by means of directors, were given in the ſame way, and acroſs the jaw, near near the articulation. Theſe alſo proving unſucceſsful, the ſhocks were next applied through the whole courſe of the ſpine. When ſhe had received about half a dozen ſhocks, in this way, a profuſe perſpiration took place, and in the ſpace of eight or ten minutes her mouth, which had been cloſed eighteen hours, opened almoſt inſtantaneouſly, and ſhe regained her ſpeech.— For ſome days after this there was a diſpoſition to tetanus ſtill prevailing, though the jaw did not again become completely locked; electricity was therefore occaſionally had recourſe to for the ſpace of a week; tonic remedies (but without the bark) were likewiſe adminiſtered, and a plaſter compoſed of empl. ſtomach. opium, and camphor, was applied to the region of the ſtomach. Under this mode of treatment the fits gradually went off, and ſhe regained, at the end of about ſix weeks from the time I firſt attended her, a perfect ſtate of health.

A period of near two years having elapſed, ſhe was again ſeized, on the 13th of June, 1790, with a fit exactly ſimilar to that which had taken place on the 7th of May, 1788, and the day following her jaw became rigid, and completely locked. As I was from home at this time, my friend Mr. Martin again gave her his aſſiſtance.

The account he gave me of her ſituation was, that he found her in a ſtate of ſtupor, with her countenance diſtorted, her head drawn backwards, and her whole frame ſtrongly convulſed.

Being unable to get his large cylindrical machine, he was obliged to employ a ſmall globe one, and a Leyden phial containing about a ſquare foot of coating; with theſe he paſſed ſeveral of the ſtrongeſt ſhocks he could well collect from the phial through her legs and arms, and in the direction of the ſpine, for near an hour, by which means the contraction of her limbs went off, and ſhe recovered her ſenſes; but the ſpaſm of the jaw did not ſubſide till about half a dozen ſhocks had been ſent through it by means of a director fixed under each ear. She was then put to bed, but before ten minutes had elapſed her jaw again became locked, and was again relieved by the application of three more ſhocks, paſſed in the ſame direction as the former ones. In this manner the tetanus continued to return and to be relieved for ſeveral hours, till at length the ſlighteſt ſhock was found ſufficient to produce the effect of opening her mouth.

My abſence from home prevented me from ſeeing her till two days afterwards. I then found that the electricity had been occafionally repeated, on the recurrence of the fits, which had now almoft totally fubfided. Tonic remedies were adminiftered, and the patient remained free from any fpafmodic complaint for a fortnight. At the end of that time, in confequence of fatigue, fhe had a return of the fits, and her jaw again became locked, and remained fo for feveral hours, till the fame remedy, the good effects of which fhe had fo repeatedly experienced, was again had recourfe to. Strong fhocks were paffed acrofs the jaw and down the fpine, as before, and by thefe means, in about twenty minutes, the fpafm was removed. After this laft attack fhe gradually recovered, and has remained well ever fince.

This cafe feems to be worthy of being recorded, both on account of the uncommon fymptoms that attended it, and of the relief obtained in it from electricity.

M. Sauvages, in his *Nofologia Methodica*, has arranged, among his fpecies, a *catalepfis hyfterica* and a *tetanus hyftericus*; but of neither of thefe has he been able to collect more than a fingle inftance; fo that they muft be confidered as rare affections; and their combination, as in the the cafe I have related, is probably a ftill more uncommon occurrence.

M. Lieutaud * fuppofes that what has been called catalepfy is conftantly an hypochondriacal or hyfterical affection. That it was fo in the cafe of my patient I am very ready to allow; but how far his opinion is applicable to other cafes of catalepfy, as defcribed by different writers, I fhall not at prefent attempt to determine.

* Precis de la Medecine pratique, Tome I. p. 302, 8vo. Paris, 1777.

